# Acute Abdominal Situations as Presenting or Flaring Manifestations of Systemic Lupus Erythematosus: A Case Series

**DOI:** 10.31138/mjr.33.3.339

**Published:** 2022-09-30

**Authors:** Polina Pavli, Ourania Gioti, Themistoklis N. Spyridopoulos, George Katsikas, George Tsourous, Antonia Elezoglou, Katerina Kaziani, Antonis Fanouriakis

**Affiliations:** 1 Third Department of Internal Medicine, Evaggelismos General Hospital, Athens, Greece,; 2 Department of Rheumatology, “Asklepieion” General Hospital, Athens, Greece,; 3 2^nd^ Unit of Diagnostic and Interventional Radiology, National and Kapodistrian University of Athens, Medical School, Evgenidion Hospital, Athens, Greece,; 4 Department of Rheumatology, Evaggelismos General Hospital, Athens, Greece,; 5 Department of Internal Medicine, “Mediterraneo” Hospital, Athens, Greece

**Keywords:** systemic lupus erythematosus, lupus peritonitis, lupus enteritis, mesenteric vasculitis, gastrointestinal involvement

## Abstract

Systemic lupus erythematosus (SLE)is a multisystem autoimmune disease, characterized by clinical heterogeneity, ranging from mild to severe, life-threatening manifestations. Although gastrointestinal (GI) symptoms are frequently encountered during disease course (mainly associated with complications of medication or infection), primary GI involvement due to SLE is rare. Among variable presentations, lupus abdominal serositis (defined as peritonitis if accompanied by symptoms and signs of acute abdomen) and lupus enteritis/mesenteric vasculitis are causes of SLE-related acute abdominal pain. They occur, although not always, in the context of high disease activity and prompt diagnosis and treatment is necessary due to their potential severe complications. However, the diagnosis of these manifestations remains challenging even for experts, especially in cases of “organ-dominant” lupus flares. Exclusion of these rare manifestations from classification criteria increases the likelihood of misdiagnosis and highlights the inherent limitations of classification criteria when the latter are used for diagnosis. Urgent abdominal computed tomography can lead to a prompt diagnosis of these lupus manifestations, especially characteristic for lupus mesenteric vasculitis. Herein, we describe four cases of patients with lupus flare, presenting with acute abdominal manifestations and highlight the potential complexity of diagnostic approach in such situations.

## MAIN POINTS

1. Although rare, primary SLE gastrointestinal involvement, including lupus peritonitis and lupus enteritis/mesenteric vasculitis, should be suspected in SLE patients presenting with abdominal pain.
2. The threshold for an abdominal CT should be low, as typical imaging findings (such as target sign and comb sign) are suggestive for the diagnosis.
3. Early diagnosis and treatment of these entities is crucial, in order to avoid severe complications (eg, ischemia or perforation).

## INTRODUCTION

Systemic lupus erythematosus (SLE) is a systemic autoimmune disorder with a wide range of clinical presentations involving almost all organs. Gastrointestinal (GI) symptoms are not uncommon in SLE and are usually related to adverse events of treatment or infectious causes. More rarely, acute abdominal situations may represent GI involvement of the disease *per se*.^1–3^ The British Isles Lupus Assessment Group (BILAG) has provided different definitions for presentations of acute abdominal pain due to SLE, based on clinical and imaging findings.^4^ Abdominal serositis occurs mainly in patients with active disease and usually responses adequately to glucocorticoids.^5^ Abdominal serositis presenting as acute abdomen with rebound tenderness/guarding constitutes lupus peritonitis. Finally, lupus mesenteric vasculitis (also known as lupus enteritis), is a rare, yet most serious cause of acute abdominal pain in SLE patients, potentially leading to bowel necrosis or perforation.^6^ Typical computed tomography (CT) findings of target sign (bowel wall thickening with abnormal wall enhancement) and comb sign (engorgement of mesenteric vessels) are important for a prompt diagnosis.

As these disease-related manifestations may result in even life-threatening complications, early diagnosis and treatment is crucial.^7^ Of note, the rarity of severe abdominal involvement in SLE has excluded these manifestations from all sets of classification criteria.^8–10^ Importantly, although the aim of classification criteria is the inclusion of patients in clinical trials, they are nevertheless frequently used for diagnostic purposes, especially by physicians with limited experience in lupus.

We herein describe four cases of SLE, presenting or flaring as acute abdomen and discuss relevant issues regarding the need for prompt diagnosis, especially when this is the sole manifestation of the disease (**Table 1**).

**Table 1.** Clinical, laboratory features and treatment of patients.

	**Case 1**	**Case 2**	**Case 3**	**Case 4**
**Age(years)/Sex**	30/F	26/F	40/F	40/F
**SLE duration(years)**	0	10	0	20
**Treatment at flare**	None	5mg PZ HCQ, MTX	None	5mg PZ HCQ
**Extra-GI manifestations at flare**	Fever, arthritis, pleuritis, pericarditis, cytopenias	Fever, arthritis, pleuritis, cytopenias	Fever, pleuritis	None
**Abnormal blood tests**	Hb 6.6g/dl WBC 2530/μl CRP 9.4mg/dl ESR 120mm/hr	Hb 10g/dl WBC 3020/μl PLT 127000/μl ALT 439 U/l AST 824 U/l CRP 22 mg/dl ESR 56 mm/hr	Hb 11.6g/dl	None
**Serological activity**	Yes	Yes	Yes	Yes
**Treatment administeredv**	1mg/kg PZ HCQ	3gr MP iv 0.6mg/kg PZ Belimumab	3gr MP iv 0.8mg/kg PZ AZA, HCQ	3gr MP iv 1mg/kg PZ CYC iv
**Follow-up**	No relapse	No relapse	No relapse	No relapse

F: female; PZ: prednisone; HCQ: hydroxychloroquine; MTX: methotrexate; GI: gastrointestinal; iv: intravenous; MP: methylprednisolone; AZA: azathioprine; CYC: cyclophosphamide

## CASE DESCRIPTIONS

### Case 1

A 30-year-old Caucasian woman with a history of Sjögren’s syndrome was referred due to a 20-day history of fever and worsening abdominal pain that appeared two days ago. She had delivered her second child by caesarean section one month before. During pregnancy, she was receiving treatment with low molecular weight heparin and low-dose prednisone for one month for unclear reasons. Sjögren’s syndrome had been diagnosed six years ago, based on polyarthritis and positive anti-Ro/anti-La, and had been treated with glucocorticoids and hydroxychloroquine for a short period.

**Figure 1.**
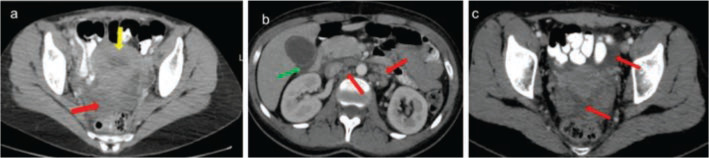
Abdominal CT images of patients 1 and 2. (**a**) Patient 1, axial CECT image: Fluid collection at the lower pelvis (red arrow) and mild fluid collection in the uterine cavity (yellow arrow). (**b**) Patient 2, axial CECT image: Pericholecystic fluid collection (green arrow); enlarged lymph nodes (red arrows). (**c**) Patient 2, axial CECT image: Fluid collection in the lower pelvis.

On admission, the patient was febrile and physical examination was remarkable for guarding and rigidity of the lower abdomen, and peripheral enema. Blood tests revealed anaemia and leukopenia (Hb=6,6g/dl, WBC=2.53x10.000), raised erythrocyte sedimentation rate (ESR=120mm/hr) and C-reactive protein (CRP=9.4mg/dl). Urgent abdominal CT scan showed fluid collections perihepatically and in the lower pelvis (**Figure 1A**).

Empirical antimicrobial treatment was initially started for a possible postoperative intraabdominal infection, without clinical improvement. A transvaginal ultrasound did not reveal findings consistent with local infection. On the third day of hospitalization, the patient developed arthralgias and thoracic pain. Physical examination revealed symmetrical polyarthritis and clinical signs of pleuritis (diminished breath sounds). New onset Raynaud’s phenomenon was also present, along with progressive worsening of leukopenia and lymphopenia. Chest CT revealed right pleural and small pericardial fluid collections. At this point, clinical manifestations were considered as possibly related to a SLE flare (fever, arthritis, polyserositis, cytopenias) and the patient was started on prednisone (1mg/kg/daily with gradual tapering) and hydroxychloroquine, while antibiotics were interrupted. Immunological testing showed diffuse hypergammaglobulinemia, positive anti-nuclear antibodies (ANA> 1: 640), anti-dsDNA >95.0 U/ml (cut-off: 7 U/ml), positive anti-Ro and anti-La, as well as IgM-anticardiolipin antibodies (52.2, normal values: 0.1–20 MPL). Complement levels were low-normal.

Following initiation of immunosuppressive therapy, the patient gradually improved in terms of clinical and laboratory findings. She was discharged in good clinical condition, with a final diagnosis of multiorgan SLE flare, initially manifesting as abdominal serositis/peritonitis. During follow-up and after having reduced glucocorticoids to acceptable daily doses, the patient remains clinically quiescent.

### Case 2

A 26–year–old Caucasian woman was admitted to another hospital with fever (up to 39^o^C) accompanied by rigor, fatigue, headache, and nausea; symptoms had started four days before admission. The patient had been diagnosed with SLE ten years ago, based on arthritis, alopecia, pleuritis and compatible serology (positive ANA and ds-DNA, hypocomplementemia). Her treatment included low-dose prednisone, hydroxychloroquine, and methotrexate.

On admission, the patient was febrile (39.6^o^C) and in distress, with mild tachycardia (110 beats/minute). Initial physical examination was remarkable only for late inspiratory crackles at both lung bases. Laboratory tests revealed normochromic anaemia and increased inflammatory markers (CRP 22 mg/dl, ESR 56 mm/hr).

On the second day of hospitalization, the patient developed progressively worsening epigastric pain with signs of guarding and rebound tenderness. Urgent CT scans showed mild bilateral pleural effusions with areas of ground glass in the lung bases, hepato- and splenomegaly, as well as fluid accumulation in the pericholecystic, perihepatic, perisplenic, and lower pelvic area (**Figure 1B,C**). Evidence of a hypodense tissue surrounding the celiac artery and portal vein was also suspected. Paracentesis of the ascitic fluid showed lymphocytic pleocytosis (350 cells, 60% lymphocytes), with negative cultures and cytology.

Broad-spectrum antibiotic treatment was administered; nevertheless, on day 3 of hospitalisation, she experienced severe clinical deterioration with hemodynamic instability and hypoxemia. Complete blood count revealed pancytopenia, without evidence of microangiopathic haemolytic anaemia, and profoundly elevated liver enzymes (AST: 824 U/l, ALT: 439 U/l), which were attributed to possible liver hypoperfusion (shock liver). Repeat CT showed deterioration of pleural and peritoneal effusions and pericholecystic enema. The patient was admitted to the Intensive Care Unit and aggressive intravenous fluid resuscitation together with enhanced antibiotics were administered. A thorough work-up for infectious causes, including bacteria and viruses, was negative. Ultrasound of the splenoportal axis and magnetic resonance cholangiopancreatography were unremarkable.

Rheumatologic evaluation at the time detected symmetrical polyarthritis of hands and feet and consulted for a possible SLE flare, presenting with cytopenias, pleuritis and peritonitis. Immunology testing was positive for ANA 1/640, anti-dsDNA (43 U/ml), lowC3 (0.77 gr/lt) and a positive direct Coombs test (++).Three daily intravenous pulses of 1gr methylprednisolone were initiated, followed by 40mg prednisone (0.6 mg/kg), leading to an immediate clinical and laboratory response. Ultimately, the patient was discharged with gradual tapering of glucocorticoids and subcutaneous belimumab was initiated; methotrexate was discontinued due to the markedly elevated liver function tests. One year later, the patient remains in clinical remission, with persistent serologic activity, but no new flares.

### Case 3

A 40-year-old Caucasian woman with a history of leukopenia and photosensitivity but no formal diagnosis of an autoimmune rheumatic disease, presented with new-onset abdominal pain, nausea, vomiting, accompanied by low grade fever. The symptoms had started 3 days prior to admission, although the patient reported recurrent episodes of abdominal pain in the preceding 5 months, without having sought medical attention.

**Figure 2.**
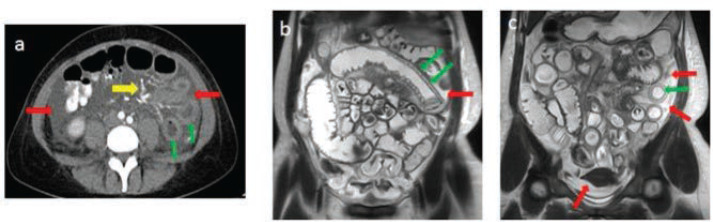
Abdominal CT and MR images of patient 3. (**a**) Axial CTA image; Ascites (red arrows); wall thickening of jejunal arterial branches (yellow arrow); jejunal wall thickening (green arrows). (**b**) MRI T2 haste coronal image: Jejunal wall thickening (green arrows); ascites (red arrow). (**c**) MRI T2 haste coronal image: Jejunal wall thickening (green arrow); ascites (red arrows).

On admission, the patient was ill-appearing and physical examination revealed metallic bowel sounds, rebound tenderness, and shifting dullness (indicative of ascites). Laboratory tests showed mild, normocytic, normochromic anaemia (white blood cells were normal). An abdominal CT scan confirmed the presence of ascites, together with profound, segmental dilatation and wall thickening of the small intestine, concerning mainly the jejunum. Paracentesis of the ascitic fluid showed pleocytosis (170 cells/μl, 55% lymphocytes), with negative cultures and cytology. Subsequent abdominal CT angiography revealed remarkable jejunal wall thickening with abnormal enhancement (target sign) and wall thickening of the jejunal branches of the superior mesenteric artery. The latter findings were also confirmed by abdominal MRI (**Figure 2**). Collectively, imaging findings were suggestive of mesenteric vasculitis. A chest CT showed bilateral pleural effusions.

Gastrointestinal endoscopy revealed segmental mucosal oedema of the duodenum and jejunum; biopsies were negative for malignancy. Following sterile blood and ascitic fluid cultures, broad-spectrum antibiotics, initiated empirically on admission, were gradually discontinued. Immunologic testing revealed positive ANA 1/320, anti-dsDNA (40 U/ml) and anti-Ro, and significantly reduced C3/C4 levels; antiphospholipid and anti-neutrophil cytoplasmic antibodies (ANCAs) were negative.

A diagnosis of SLE was suspected following rheumatologic evaluation, based on both clinical manifestations (mesenteric vasculitis/peritonitis, history of photosensitivity and leukopenia) and compatible serology. Three daily intravenous methylprednisolone pulses 1 gr were administered, followed by 50 mg prednisone orally. Azathioprine and hydroxychloroquine were also started. The patient experienced a gradual clinical and imaging improvement. Glucocorticoids were ultimately discontinued six months later and over 4-year follow-up the patient remains in glucocorticoid-free sustained remission.

### Case 4

A 40-year-old Caucasian woman with a 20-year history of SLE (based on arthritis, rash, positive ANA, anti-dsDNA, anti-Sm, anti-Ro and hypocomplementemia), was admitted to another hospital due to epigastric pain and vomiting. Prior to admission, she was treated with hydroxychloroquine and low-dose prednisone, and was clinically quiescent, although serologically active.

**Figure 3.**
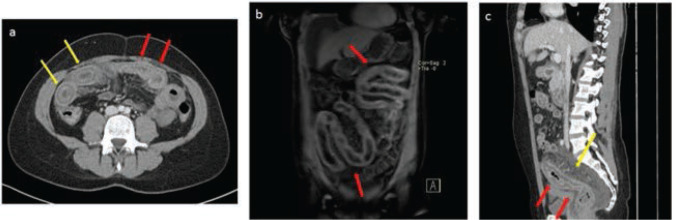
Abdominal CT, MR enteroclysis images of patient 4. (**a**) Axial CT image; jejunal wall thickening, target sign (red arrows); wall thickening of the ileum (yellow arrows). (**b**) MR enteroclysis image; wall thickening of jejunum and ileum (red arrows). (**c**) Lateral CT image; wall thickening of sigmoid and rectum (red arrows); ascites (yellow arrow).

Clinical examination upon admission revealed guarding, rebound tenderness and decreased bowel sounds. Blood tests were unremarkable with normal inflammatory markers; complement levels were low and anti-dsDNA significantly elevated. Urgent abdominal CT revealed ascites and bowel wall thickening with a typical target sign, mainly concerning the jejunum and a smaller part of the ileum. Multiple enlarged lymph nodes were also present, and the findings were confirmed by MR enteroclysis (**Figure 3**). Upper GI endoscopy showed no abnormalities and histological examination of small intestine showed non-specific inflammatory mucosal lesions. A thorough work-up for infectious causes, including *Yersinia* and cytomegalovirus, was negative. Antibiotics were initially started empirically, but based on patient’s medical history and imaging findings, a diagnosis of lupus enteritis/mesenteric vasculitis was suspected, and high-dose prednisone (1mg/kg daily) was added. The patient showed a clinical improvement and was transferred to our hospital for further management.

On day 2 of hospitalization and following reduction of prednisone dose, the patient experienced recurrence of abdominal pain and vomiting, together with rebound tenderness in examination. Repeat abdominal CT and CT angiography revealed improvement of findings concerning the small intestine, but a profound dilatation and wall thickening of the transverse colon, sigmoid and rectum, without signs indicative of thrombosis or ischemia. Ascitic fluid analysis showed normal white cell count and culture was negative.

With a presumed lupus flare presenting with lupus enteritis, three pulses of 1gr methylprednisolone were administered, followed by 0.6mg/kg prednisone orally. Intravenous cyclophosphamide was also started, due to the patient’s deterioration after initial glucocorticoid tapering. The patient showed an immediate clinical response and subsequent ultrasound revealed improvement of bowel dilatation. Three months later, the patient is asymptomatic, on treatment with monthly pulses of cyclophosphamide and gradual prednisone tapering.

## DISCUSSION

GI symptoms are frequently encountered in SLE patients and may vary from minor and non-specific, such as nausea, vomiting, diarrhoea, to more serious like malabsorption, ascites or acute abdomen. Although usually related to medication adverse effects, infections or metabolic disorders, abdominal manifestations may occasionally represent disease-related involvement. A wide spectrum of clinical entities including abdominal serositis/peritonitis, mesenteric vasculitis, intestinal pseudo-obstruction, protein-losing enteropathy, have been described as clinical manifestations of abdominal lupus. GI involvement is usually seen in the context of multisystem disease activity, but infrequently may appear as an isolated or even presenting symptom of the disease. To this end, physicians’ awareness for primary acute lupus abdominal situations is crucial, because if remain unrecognised, they can lead to severe morbidity.^2^ Although pleuritis and pericarditis are cardinal findings in SLE, ascites as a clinical manifestation of lupus serositis is rare and usually mild, with exudative characteristics (SAAG<1.1) and a lymphocytic predominance. However, cases of massive painless ascites as a prominent and occasionally initial manifestation of lupus serositis have been reported.^11,12^ When accompanied by abdominal pain and clinical signs of peritoneal irritation, the condition is usually termed lupus peritonitis (BILAG-2004 glossary). The latter may appear as acute surgical abdomen frequently leading to misdiagnosis.^3^ Pathophysiologically, peritoneal microvessel inflammations considered the most probable underlying mechanism. Immune-complex deposition and complement activation have been described in the peritoneal microvasculature, together with granular depositions of IgG and complement along the mesothelial layer and blood vessels in SLE patients with serositis.^13^

The terms lupus enteritis and mesenteric vasculitis are often used interchangeably, and present with non-specific symptoms, such as abdominal pain, vomiting, diarrhoea and fever.^6^ They usually -although not always-manifest in the context of generalized disease activity.^2^ Pathophysiologically, both inflammatory and thrombotic pathogenetic mechanisms have been proposed. The former is a classic leucocytoclastic vasculitis due to immune-complex deposition in vessel walls and complement activation. The thrombotic form is caused by vascular thrombosis due to circulating antiphospholipid antibodies (aPLs), a finding not confirmed in patients 3 and 4 of our series.^14,15^ Since different bowel wall layers are not equally sensitive to diminished blood supply, variable clinical symptoms may be present, depending on the layer predominantly involved. Ischemia of mucosal blood vessels leads to ulceration, diarrhoea, and haemorrhage, while deeper involvement of the bowel wall can be manifested as pseudo-obstruction, perforation and ultimately peritonitis.^14^

Computed tomography is the gold standard for the diagnosis of mesenteric vasculitis, showing focal or diffuse bowel wall thickening with abnormal wall enhancement (target sign), characteristic dilated bowel loops, mesenteric vessel stenosis and engorgement (comb sign) and increased attenuation of the mesenteric fat; these findings were evident in cases 3 and 4 of our series.^16^ Furthermore, ultrasonography is an underestimated tool, that can be helpful in diagnosis and follow-up of mesenteric vasculitis, showing bowel oedema, whereas the “thumb-print” sign is a radiographic finding indicative of ischemic changes, caused by irregular bowel fold thickening. Endoscopy may reveal signs of either ischemia or ulcers that look like “punch out” mucosal areas. Although lupus enteritis/mesenteric vasculitis are not typically diagnosed by histological biopsy due to the location of affected vessels that are usually inaccessible, histology is nevertheless important to rule out competing diagnoses.^14^

There are limited data guiding therapeutic approach to these rare lupus manifestations, as controlled studies are lacking. Moderate to high glucocorticoid doses (usually following intravenous pulses), are considered as first-line therapy, leading to rapid response in most patients. In severe or refractory cases (like patients 2–4 in our series), or in relapses, immunosuppressive agents should be considered.^5,17,18^ The choice of drug is based on severity of manifestation or concurrent disease activity from other organ-systems(renal, neuropsychiatric).^6^ Case 4 demonstrates the necessity of cyclophosphamide in a severe lupus abdominal flare refractory to glucocorticoids, whereas in case 2 belimumab was preferred because of extra-gastrointestinal (musculoskeletal) and persistent serological activity.

Patients 3 and 4 in our case series highlight the challenging scenario of “organ-dominant” lupus. Both patients presented with a major lupus abdominal manifestation, lacking other significant clinical findings; in patient 3, particularly, this was the presenting manifestation leading to SLE diagnosis (although history of photosensitivity and leukopenia were “alarming” findings).These patients represent a true diagnostic challenge, which becomes greater if a diagnosis of SLE does not pre-exist. Classification criteria are not helpful in this regard, because abdominal serositis and mesenteric vasculitis (owing to their rarity) are not included in any set of criteria (serositis is defined as pericardial or pleural inflammation). These situations highlight the inherent limitation of classification criteria, which by default aim for a high specificity and inevitably have to exclude rare manifestations of diseases.^19^ Lacking diagnostic criteria, the diagnosis of SLE will continue to rely on expert physician judgment, however physicians with less experience in the disease should also keep a high index of suspicion in patients presenting with even solitary organ-threatening manifestations.

In conclusion, we describe four patients with acute abdominal manifestations as presenting or predominant symptoms of a lupus flare. Rather than being distinct entities, lupus abdominal manifestations could be considered as a clinical continuum with variable grade of severity, sharing same pathophysiological mechanisms and bearing overlapping symptoms and imaging findings.

## References

[1] Alves SC Fasano S Isenberg DA. Autoimmune gastrointestinal complications in patients with systemic lupus erythematosus: case series and literature review. Lupus 2016 Dec;25(14):1509–19. 27329649 10.1177/0961203316655210

[2] Brewer BN Kamen DL. Gastrointestinal and Hepatic Disease in Systemic Lupus Erythematosus. Rheum Dis Clin N Am 2018 Feb;44(1):165–75. 10.1016/j.rdc.2017.09.011 PMC5796428 29149925

[3] Li Z Xu D Wang Z Wang Y Zhang S Li M Gastrointestinal system involvement in systemic lupus erythematosus. Lupus 2017 Oct;26(11):1127–38. 28523968 10.1177/0961203317707825

[4] Yee C-S Farewell V Isenberg DA Prabu A Sokoll K Teh L-S Revised British Isles Lupus Assessment Group 2004 index: A reliable tool for assessment of systemic lupus erythematosus activity. Arthritis Rheum 2006 Oct;54(10):3300–5. 17009266 10.1002/art.22162

[5] Man BL Mok CC. Serositis related to systemic lupus erythematosus: prevalence and outcome. Lupus. 2005 Oct;14(10):822–6. 16302677 10.1191/0961203305lu2187oa

[6] Janssens P Arnaud L Galicier L Mathian A Hie M Sene D Lupus enteritis: from clinical findings to therapeutic management. Orphanet J Rare Dis 2013;8(1):67. 23642042 10.1186/1750-1172-8-67 PMC3651279

[7] Tian X-P Zhang X. Gastrointestinal involvement in systemic lupus erythematosus: Insight into pathogenesis, diagnosis and treatment. World J Gastroenterol 2010;16(24):2971–7. 20572299 10.3748/wjg.v16.i24.2971 PMC2890936

[8] Hochberg MC. Updating the American college of rheumatology revised criteria for the classification of systemic lupus erythematosus. Arthritis Rheum 1997 Sep;40(9):1725–1725. 10.1002/art.1780400928 9324032

[9] Petri M Orbai A-M Alarcón GS Gordon C Merrill JT Fortin PR Derivation and validation of the Systemic Lupus International Collaborating Clinics classification criteria for systemic lupus erythematosus. Arthritis Rheum 2012 Aug;64(8):2677–86. 22553077 10.1002/art.34473 PMC3409311

[10] Aringer M Costenbader K Daikh D Brinks R Mosca M Ramsey-Goldman R 2019 European League Against Rheumatism/American College of Rheumatology classification criteria for systemic lupus erythematosus. Ann Rheum Dis 2019 Sep;78(9):1151–9. 31383717 10.1136/annrheumdis-2018-214819

[11] Forouhar-Graff H Dennis-Yawingu K Parke A. Insidious onset of massive painless ascites as initial manifestation of systemic lupus erythematosus. Lupus 2011 Jun;20(7):754–7. 21335398 10.1177/0961203310386275

[12] Geraldino-Pardilla L Gartshteyn Y Mendoza F. Massive Serositis as the Initial Presentation of Systemic Lupus Erythematosus: A Report of Two Cases and Review of the Literature. J Rheum Dis Treat 2015;1(4):028.

[13] Bitran J McShane D Ellman MH. Ascites as the major manifestation of systemic lupus erythematosus. Arthritis Rheum 1976;19(4):782–5. 942508 10.1002/1529-0131(197607/08)19:4<782::aid-art1780190421>3.0.co;2-i

[14] Ju JH Min J-K Jung C-K Oh SN Kwok S-K Kang KY Lupus mesenteric vasculitis can cause acute abdominal pain in patients with SLE. Nat Rev Rheumatol 2009 May;5(5):273–81. 19412194 10.1038/nrrheum.2009.53

[15] Cervera R Espinosa G Cordero A Oltra MR Unzurrunzaga A Rossiñol T Intestinal Involvement Secondary to the Antiphospholipid Syndrome (APS): Clinical and Immunologic Characteristics of 97 Patients: Comparison of Classic and Catastrophic APS1. Semin Arthritis Rheum 2007 Apr;36(5):287–96. 17207521 10.1016/j.semarthrit.2006.09.003

[16] Malaviya AN Sharma A Agarwal D Kapoor S Garg S Singh S Acute abdomen in SLE: Acute abdomen in SLE. Int J Rheum Dis 2011 Feb;14(1):98–104. 21303489 10.1111/j.1756-185X.2010.01581.x

[17] Lee C-K Ahn M S Lee E Y Shin J H Cho Y-S Ha H K Acute abdominal pain in systemic lupus erythematosus: focus on lupus enteritis (gastrointestinal vasculitis). Ann Rheum Dis 2002 Jun 1;61(6):547–50. 12006332 10.1136/ard.61.6.547 PMC1754133

[18] Lian T-Y Edwards CJ Chan S-P Chng H-H. Reversible acute gastrointestinal syndrome associated with active systemic lupus erythematosus in patients admitted to hospital. Lupus 2003 Aug;12(8):612–6. 12945720 10.1191/0961203303lu433oa

[19] Bertsias GK Pamfil C Fanouriakis A Boumpas DT. Diagnostic criteria for systemic lupus erythematosus: has the time come? Nat Rev Rheumatol 2013 Nov;9(11):687–94. 23838616 10.1038/nrrheum.2013.103

